# The first complete mitochondrial genome sequence of the common Baya weaverbird (*Ploceus philippinus*) from southern India

**DOI:** 10.1080/23802359.2025.2457454

**Published:** 2025-02-04

**Authors:** Venkatesh Nagarajan-Radha, Subanithi-Purnima Murugan, Paramanantha Swami Doss Devaraj

**Affiliations:** ^a^ School of Life and Environmental Sciences, Behaviour Ecology and Evolution Lab, The University of Sydney, Camperdown, Australia; ^b^ PG Research Department of Zoology, St. John’s College, Palayamkottai, India

**Keywords:** Baya weaverbird, mitochondrial genome, phylogeny, Ploceidae

## Abstract

The common Baya weaverbird, Ploceus philippinus (Linnaeus, 1766), is best known for its nest construction behaviour. Yet, no genomic studies have been conducted on this species to date. We sequenced the mitochondrial genome of P. philippinus sampled from southern India. The circular mitochondrial genome of 16,867 bp contains 13 protein-coding genes, 22 transfer RNAs, two ribosomal RNAs (12S and 16S subunits), and a non-coding control region. A maximum-likelihood phylogenetic tree analysis placed P. philippinus and P. nigricollis weaverbirds in a separate clade among other bird species. The mitochondrial genome sequence would benefit future genetic studies in weaverbirds.

## Introduction

There are over 116 species of weaverbirds (Aves: Ploceidae) found across Africa and Asia (Del Hoyo et al. 2010). Weaverbirds exhibit remarkable diversity in their song displays, nest structure, social behaviors, and plumage color (Collias and Collias 1964; Walsh et al. 2010; Street et al. 2022). Weaverbirds are particularly well known for their nest construction behavior. Male weaverbirds invest significant amounts of time and energy to build intricate nests and attract females for mating (Quader 2005). Indeed, the mating success of males depends on the quality of the nests (Quader 2006). However, whether the nest-building behavior of male weaverbirds is stochastic and whether the decisions behind the nest structure have a genetic control are unclear.

**Figure 1.**
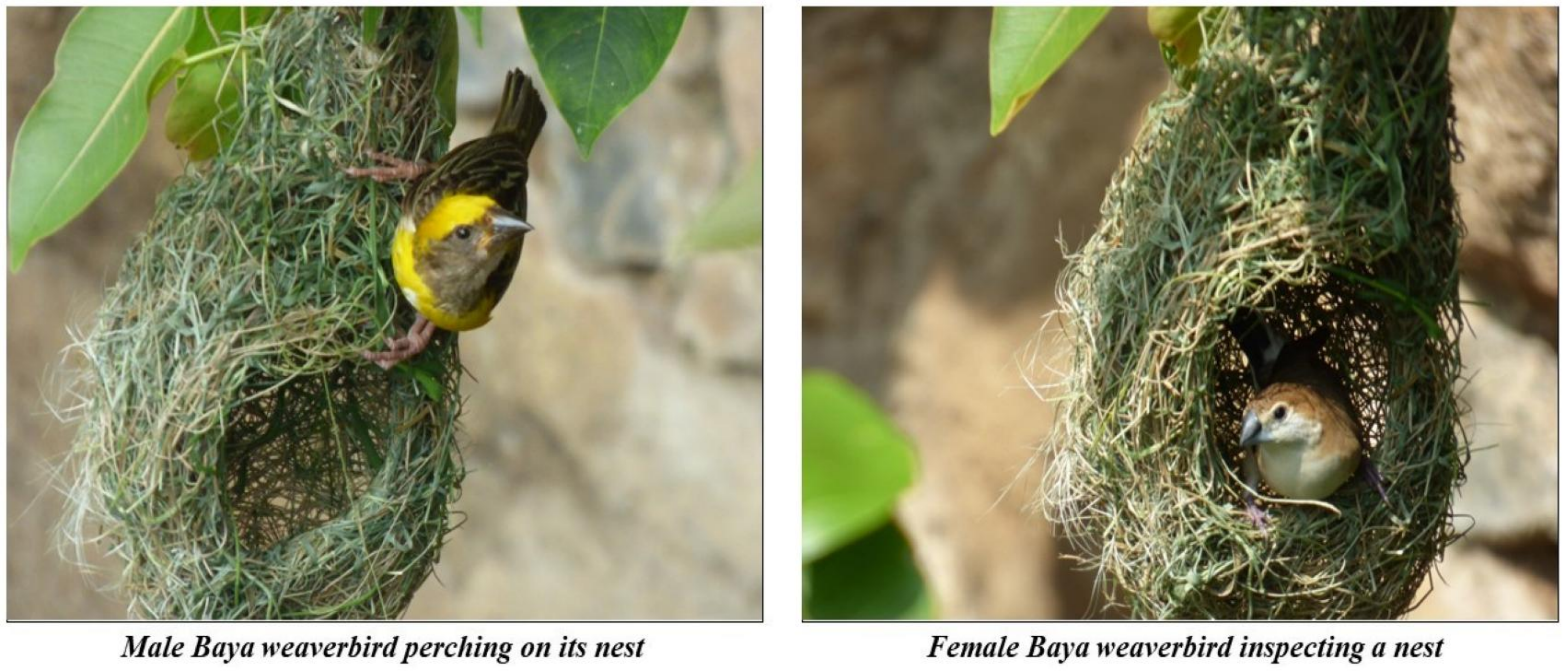
Photo of a male (left panel) and female (right panel) common Baya weaverbird, *P. philippinus*, observed in our study area (GPS coordinates: 8.69° N, 77.68° E). Photo credit: Mr VM Santhanamahalingam.

We studied *Ploceus philippinus* (subspecies: *philippinus*, NCBI: txid414895) found across the Indian subcontinent (Figure 1). Males construct nests in thorny bushes, Palmyra palm fronds and coconut tree fronds in southern India (Quader 2005, 2006). Although we understand nest construction behavior in the *P. philippinus*, no genomic research has been conducted on these weaverbirds to understand the genetic architecture of the mitochondrial genome that enables continuous energy production for nest construction during mating seasons.

## Materials and methods

We identified nesting sites of *P. philippinus* in a village in Tirunelveli district of southern Tamil Nadu, India (GPS coordinates: 8.69° N, 77.68° E). V Nagarajan-Radha, SP Murugan, and DPS Doss collected field-shed feathers and fecal pellets from beneath their nests wherever possible and transported them to the lab in sterile bags. A specimen was deposited at – the Centre for Behavioural and Immuno Ecology in St. John’s College (webpage: https://stjohnscollege.edu.in/, contact person and email: Dr D Paramanantha Swami Doss, dossanand@gmail.com) under the voucher number CBW_VGK-001.

We isolated whole genomic DNA from these samples following standard protocol for feather DNA and fecal DNA extractions. For feather DNA, we cut the feather tip with a sterile scalpel and incubated it in 500 µL lysis buffer containing Tris–HCl, ethylenediaminetetraacetic acid (EDTA) and 5% sodium dodecyl sulfate (pH 8.0). We added each tube containing the feather samples with 2 mg/mL proteinase K solution and incubated at 56 °C for digestion. The resulting lysate was directly used for column-based DNA extraction using a QIAGEN DNeasy kit (Hilden, Germany, Catalog# 69504). Similarly, we initially solubilized the fecal samples in 500 µL Tris–EDTA buffer (pH 8.0) for fecal DNA extraction. We mixed the samples on a vortexer for 30 s and filtered the samples using a sterile gauze pad packed within a 2 mL syringe. This process allowed us to filter out solid materials in the fecal samples. We centrifuged the filtrate at 15,000 rpm for 5 min and used the resulting pellet for DNA extraction with a QIAGEN DNeasy kit (Hilden, Germany). Isolated genomic DNA from both samples was solubilized in Nuclease-free water (HiMedia Labs, Modautal, Germany) and checked for quality using Nanodrop 2000 (Thermo Scientific, Waltham, MA). We pooled the DNA samples and sequenced the mitochondrial genome using 2 × 250 bp sequencing chemistry on an Illumina MiSeq platform at MedGenome Labs Ltd. (Bengaluru, India).

The raw reads (in FASTQ format) were trimmed for adaptor sequences and paired to create a unified library in Geneious Prime (v2024.0.2). We performed a *de novo* assembly of the reads to generate contigs of the desired size (∼16 kb). We simultaneously mapped the reads to the *P. nigricollis* mitochondrial reference genome sequence. The annotations for mitochondrial genes were extracted from standard mitochondrial reference genomes in NCBI – *Taeniopygia guttata*, *Gallus gallus*, and *P. nigricollis*. We built an approximate maximum-likelihood tree using the FastTree program (Price et al. 2009) in Geneious. We used only the protein-coding region of mitochondrial genome sequences to deduce the phylogenetic relationship between *P. philippinus*, *P. nigricollis*, and other closely related bird species.

## Results and discussion

**Figure 2.**
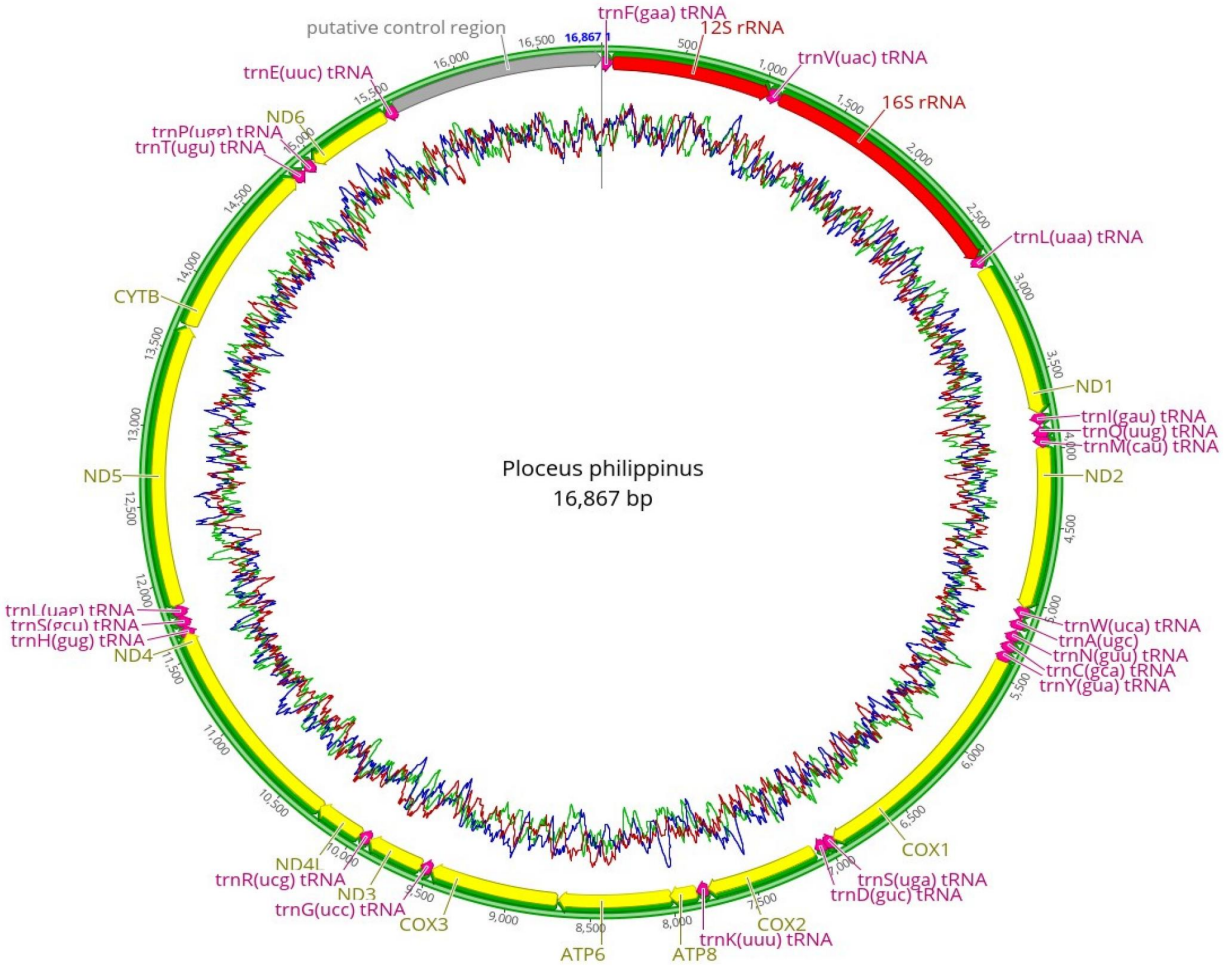
Circular view of the 16,867 bp mitochondrial genome of *P. philippinus*. The 2 × 250 bp paired-end short reads were assembled *de novo*, and gene annotations were extracted from the mitochondrial reference genome of *P. nigricollis* (NC_051038). Segments in the inner circle in red color show ribosomal RNAs, transfer RNAs in pink, protein-coding genes in yellow, and the non-coding control region in grey. The position of nucleotides is shown on the outer ring.

**Figure 3.**
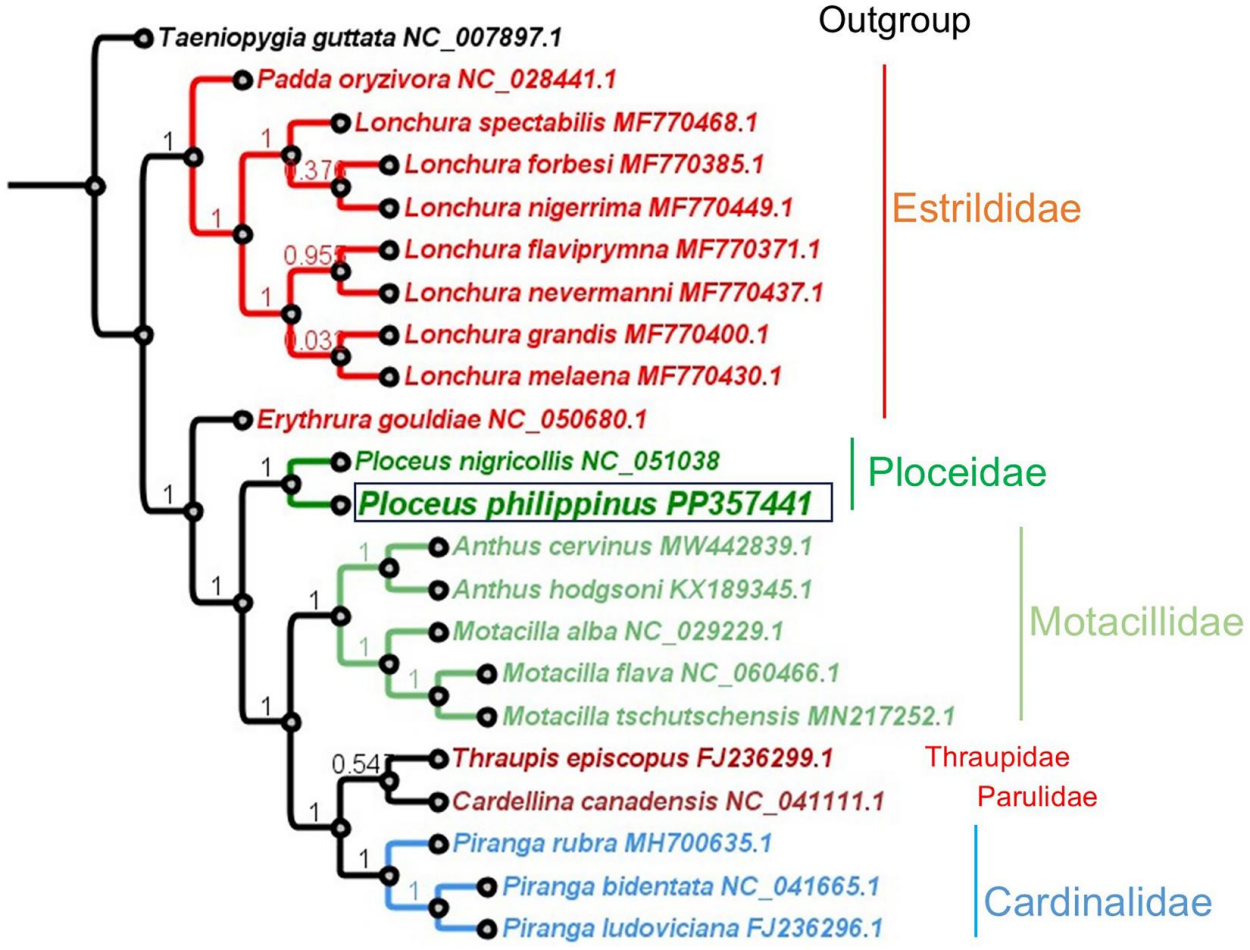
Maximum-likelihood phylogenomic tree depicting the relationship between the weaverbirds *Ploceus* sp. (highlighted in green) and bird species. The FastTree support values are specified on the branch nodes. The family and sub-family names are mentioned alongside species and corresponding GenBank accession numbers in the tree. The sequences used for phylogenetic analysis include NC_007897 (Mossman et al. 2006), NC_028441 (Huang and Zeng 2016), all sequences belonging to the *Lonchura* genus (Estrildidae family) highlighted in red (Stryjewski and Sorenson 2017), NC_050680 (Xue et al. 2020), NC_051038 (Feng et al. 2020), MW442839, KX189345 (Sun et al. 2016), NC_029229 (Dong et al. 2016), NC_060466 (Yang et al. 2022), MN217252 (Gao et al. 2019), FJ236299, NC_041111 (Coughlin et al. 2019), MH700635 (Campillo et al. 2019), NC_041665, and FJ236296.

We found the feather and fecal DNA samples to yield good coverage of the mitochondrial genome sequence of *P. philippinus* (Supplementary Figure S1). This species has a circular mitochondrial genome of 16,867 bp (Accession No: PP357441). The sequence contained 13 protein-coding genes, two ribosomal RNAs (small 12S and large 16S rRNA), 22 tRNAs, and a non-coding control region (Figure 2). We estimated the individual base composition to be 30.1% adenine, 31% cytosine, 14.7% guanine, and 24.2% thymine. We found the protein-coding genes encoded on the heavy strand (forward direction) except for the NADH subunit 6 (ND6) gene encoded on the light strand. The protein-coding sequences used ATG as the first start codon. We found 22 tRNAs of variable size, the smallest being 66 bp and the longest being 75 bp long. Eight tRNAs are encoded on the light strand and 14 tRNAs on the heavy strand. The two subunits of rRNAs were found at the beginning of the coding sequence with a length of 976 bp (12S rRNA) and 1592 bp (16S rRNA), respectively. Lastly, the non-coding control region was 1295 bp long and AT-rich. The maximum-likelihood phylogenomic tree analysis of the full-length mitochondrial genome sequences showed *P. philippinus* and the African social weaverbird *P. nigricollis* to form a separate branch, among other bird species sourced globally (Figure 3). We predict that the mitochondrial genome sequence presented here will help future studies investigating species diversity, population genetics and reproductive fitness of *P. philippinus*.

## Supplementary Material

Supplementary material.docx

## Data Availability

The mitochondrial genome sequence data has been uploaded to GenBank NCBI at https://www.ncbi.nlm.nih.gov under the accession number PP357441. The *P. philippinus* associated BioSample, BioProjects, and SRA numbers are SAMN41064439, PRJNA1103938, and SRR28904557, respectively.

## References

[CIT0001] Campillo LC, Burns KJ, Moyle RG, Manthey JD. 2019. Mitochondrial genomes of the bird genus *Piranga*: rates of sequence evolution, and discordance between mitochondrial and nuclear markers. Mitochondrial DNA B Resour. 4(2):2566–2569. doi:10.1080/23802359.2019.1637286. 33365629 PMC7687373

[CIT0002] Collias EC, Collias NE. 1964. The development of nest-building behavior in a weaverbird. Auk. 81(1):42–52. doi:10.2307/4082609.

[CIT0003] Coughlin EM, Shamblin BM, Tumas HR, Chandler RB, Nairn CJ. 2019. The complete mitochondrial genome of the Canada Warbler (*Cardellina canadensis*). Mitochondrial DNA B Resour. 4(1):450–451. doi:10.1080/23802359.2018.1555017.

[CIT0004] Del Hoyo J, Elliott A, Christie D. 2010. Handbook of the birds of the world. Weavers to New World warblers. Barcelona: Lynx Edicions.

[CIT0005] Dong X, Pan T, Kang X, Zhang Y, Sun X, Qian L. 2016. Complete mitochondrial genome of *Motacilla alba* and implications for Motacillidae taxonomy. Mitochondrial DNA A DNA Mapp Seq Anal. 27(6):4675–4676. doi:10.3109/19401736.2015.1106497. 26641012

[CIT0006] Feng S, Stiller J, Deng Y, Armstrong J, Fang Q, Reeve AH, Xie D, Chen G, Guo C, Faircloth BC, et al. 2020. Dense sampling of bird diversity increases the power of comparative genomics. Nature. 587(7833):252–257. doi:10.1038/s41586-020-2873-9. 33177665 PMC7759463

[CIT0007] Gao X, Xu D, Xia T, Dou H, Sha W, Zhang H. 2019. The complete mitochondrial genome of Eastern Yellow Wagtail (*Motacilla tschutschensis*). Mitochondrial DNA B Resour. 4(2):3486–3487. doi:10.1080/23802359.2019.1674737. 33366051 PMC7707198

[CIT0008] Huang L, Zeng B. 2016. The mitochondrial genome of white Java sparrow (*Padda oryzivora*). Mitochondrial DNA A DNA Mapp Seq Anal. 27(6):4653–4654. doi:10.3109/19401736.2015.1106487. 26644271

[CIT0009] Mossman JA, Birkhead TR, Slate JON. 2006. The whole mitochondrial genome sequence of the zebra finch (*Taeniopygia guttata*). Mol Ecol Notes. 6(4):1222–1227. doi:10.1111/j.1471-8286.2006.01497.x.

[CIT0010] Price MN, Dehal PS, Arkin AP. 2009. FastTree: computing large minimum evolution trees with profiles instead of a distance matrix. Mol Biol Evol. 26(7):1641–1650. doi:10.1093/molbev/msp077. 19377059 PMC2693737

[CIT0011] Quader S. 2005. Elaborate nests in a weaverbird: a role for female choice? Ethology. 111(12):1073–1088. doi:10.1111/j.1439-0310.2005.01134.x.

[CIT0012] Quader S. 2006. What makes a good nest? Benefits of nest choice to female Baya Weavers (*Ploceus philippinus*). Auk. 123(2):475–486. doi:10.1642/0004-8038(2006)123[475:WMAGNB]2.0.CO;2.

[CIT0013] Street SE, Jaques R, De Silva TN. 2022. Convergent evolution of elaborate nests as structural defences in birds. P R Soc B Biol Sci. 289:20221734. 10.1098/rspb.2022.1734 PMC9768638 36541171

[CIT0014] Stryjewski KF, Sorenson MD. 2017. Mosaic genome evolution in a recent and rapid avian radiation. Nat Ecol Evol. 1(12):1912–1922. doi:10.1038/s41559-017-0364-7. 29085063

[CIT0015] Sun P, Zhang C, Pang M, Qian L, Pan T, Wang H, Zhang B. 2016. The complete mitochondrial genome of *Anthus hodgsoni* (Passeriformes: Motacillidae). Mitochondrial DNA B Resour. 1(1):504–505. doi:10.1080/23802359.2016.1192507. 33490404 PMC7800981

[CIT0016] Walsh PT, Hansell M, Borello WD, Healy SD. 2010. Repeatability of nest morphology in African weaver birds. Biol Lett. 6(2):149–151. doi:10.1098/rsbl.2009.0664. 19846449 PMC2865054

[CIT0017] Xue X-M, Nan C-H, Fei Y-L, Jiang J, Chen Y-X. 2020. The complete mitochondrial genome of Gouldian finch (*Erythrura gouldiae*) and its phylogenetic analysis. Mitochondrial DNA B Resour. 5(2):1455–1456. doi:10.1080/23802359.2020.1741465.

[CIT0018] Yang C, Du X, Liu Y, Yuan H, Wang Q, Hou X, Gong H, Wang Y, Huang Y, Li X, et al. 2022. Comparative mitogenomics of the genus *Motacilla* (Aves, Passeriformes) and its phylogenetic implications. Zookeys. 1109:49–65. doi:10.3897/zookeys.1109.81125. 36762344 PMC9848870

