# Supplementary material for: The first complete mitochondrial genome sequence of the common Baya weaverbird (Ploceus philippinus) from southern India
Source: Mitochondrial DNA B Resour. 2025 Feb 4;10(3):183–6. doi: 10.1080/23802359.2025.2457454 (PMC11795757; doi:10.1080/23802359.2025.2457454)
Supplement: Supplementary material.docx [file TMDN_A_2457454_SM0552.docx]

**The first complete mitochondrial genome sequence of the common Baya weaverbird *Ploceus philippinus*, from southern India**

Venkatesh Nagarajan-Radha^1,2*^, Subanithi-Purnima Murugan^2^ and Paramanantha Swami Doss Devaraj^2^

^1^Behaviour Ecology and Evolution Lab, School of Life and Environmental Sciences, The University of Sydney, Camperdown, New South Wales 2006, Australia.

^2^PG Research Department of Zoology, St. John’s College, Palayamkottai 627002, Tamil Nadu, India.

^*^Correspondence: [venkatesh.nagarajan.radha@gmail.com](mailto:venkatesh.nagarajan.radha@gmail.com)

**Supplementary material**


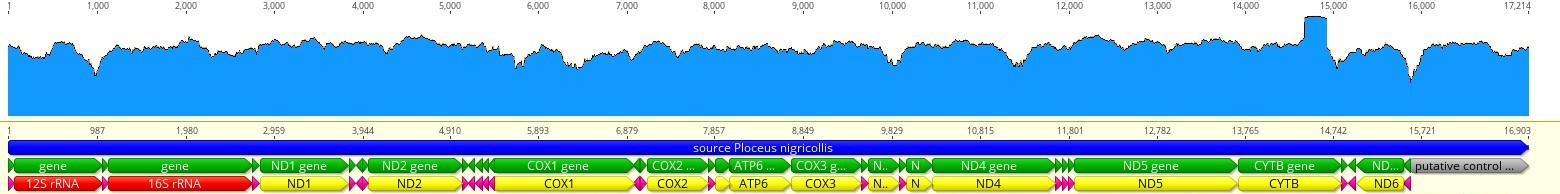


**Figure S1**. The coverage plot of short reads derived from the common Baya weaverbird (*P. philippinus*) assembled to reference mitochondrial genome of the African weaverbird *P. nigricollis* generated using Geneious Prime v2024.0.2.
